# Improving Data Integrity in Public Health: A Case Study of an Outbreak Management System in Nigeria

**DOI:** 10.9745/GHSP-D-21-00240

**Published:** 2021-11-29

**Authors:** Bosun Tijani, Tomi Jaiyeola, Busayo Oladejo, Zahra Kassam

**Affiliations:** a Co-creation Hub, Lagos, Nigeria.; b Co-creation Hub, Kigali, Rwanda.

## Abstract

The completeness and accuracy of data in the Nigerian health care system is a challenge. Studies have shown that the data quality, and by extension data integrity, has been suboptimal and thus poses a barrier to strengthening service delivery. This article showcases how the design process sparked the concept for an intervention to improve the integrity of public health data being collected in Nigeria.

In collaboration with the Nigerian Institute of Medical Research (NIMR) and Lifebank, the Co-creation Hub team conducted formative research with the coronavirus disease (COVID-19) test center managers at NIMR. The insights informed the development of the features for an outbreak management system. These features were refined through an iterative process of development and continuous feedback from the end users.

NIMR reported an improvement in its data collection process and data integrity. They reported that (1) almost all data collection by the test center was now automated, thereby minimizing the proportion of inaccurate and repeat entry in comparison to data collected in other parts of the same center; (2) the auto-validation feature of the system ensured that all required fields of a patient’s information were completed and verified, thereby ensuring 100% data completeness; and (3) the validation and verification feature ensured that patients’ contact information was validated.

The integration of this intervention into the current health information system ensures an improvement in the accuracy and validity of health care data being collected and stored.

## Abstract (precis)

Because of existing data collection and data integrity challenges in Nigeria, the COVID-19 pandemic posed an unprecedented challenge for data and its use in decision making because of the speed and scale of the necessary response. Using a human-centered design approach to co-create an outbreak management system streamlined data and sample collection and management to improve data collection and integrity.

## Teaser

**Key Messages:**

The design of an outbreak management system to automate and streamline data collection and validation at drive-through COVID-19 test centers ensured the fast and efficient data and sample collection and ensured that all data that were collected were accurate and complete.

**Key Implication:**

Integrating the automated data collection system within the current health information system in Nigeria will help address the challenge of inaccurate, incomplete, and invalid health care data being collected and stored and improve the health system’s capacity to respond to public health emergencies, such as COVID-19.

## BACKGROUND

Studies have shown that one of the most important factors in improving service delivery in health care is the quality of data.^1^ Data are a fundamental part of health care delivery and can be used for government planning and resource management and allocation. Therefore, maintaining the integrity of health care data—the accuracy, completeness, and validity of health care data being collected and stored—is integral to quality health care provision.

Over the years, health care facilities in low- and middle-income countries like Nigeria have struggled to maintain complete, accurate, and valid patient data. This challenge can be attributed to factors such as inadequate manpower, lack of technical knowledge, and an increasing volume of patients.^2^

With the coronavirus disease (COVID-19) pandemic rapidly crossing borders and spreading across countries and continents, leading to the death of millions of people and hospitalization of more, health systems were overwhelmed across the globe. The novelty of COVID-19 drove an urgency in the process of collecting, assessing, and analyzing all available data to gather insight on the scope and scale of the pandemic while also providing insight into how the disease is spreading and the impact of interventions in place to manage the pandemic. Thus, the need for collecting and maintaining quality data has become even more important.

To support Nigeria’s COVID-19 testing capacity, the Nigerian Institute of Medical Research (NIMR) established a drive-through test center^3^ in Lagos, Nigeria, to conduct free testing. The test center can process sample collection at a quicker pace, thereby allowing a higher number of cases to be tested daily in addition to other local clinics. The data collected at the test center as well as from other sources were to be evaluated for research and to inform policies for managing the pandemic,^4^ therefore, the data had to be of high quality.

In April 2020, Co-creation Hub (CcHUB) embarked on a collaborative research and development project with NIMR and Lifebank, a health care technology and logistics company, to study the test center’s data collection system, identify gaps, and find ways to support or strengthen the test center’s data collection capacity with the use of design and technology. The team used a human-centered design (HCD) approach to understand the problem and co-create a viable solution to this challenge.

During the past decade, there has been an increasing adoption and integration of design thinking and practices in development initiatives, particularly in global health programming.^5^ The Design for Health^6^ community—launched by the Bill & Melinda Gates Foundation and the Center for Innovation and Impact in the United States Agency for International Development’s (USAID) Bureau for Global Health—defined 3 models by which design methods and mindsets can be applied to developing global health solutions: spark, ingredient, or end-to-end.

In this article, we discuss how we used design as a “spark” of inspiration to reframe the understanding of the existing data-related problems at the test center, develop new ideas and innovations, and push boundaries to think differently.

We discuss how we used design as a “spark” of inspiration to reframe the understanding of the existing data-related problems at the test center, develop new ideas and innovations, and push boundaries to think differently.

## USING DESIGN AS THE SPARK

In Nigeria, health care processes have historically been using paper-based systems, and the mindset around rethinking the use of technology posed a big barrier when thinking about solving problems. COVID-19 presented an unprecedented challenge for data collection and its use in decision making due to the disease’s novel nature, the speed and scale of the necessary pandemic response, and the existing data integrity issues. Initially, the center was using a paper-based record system, which raised questions about the data integrity and quality.

The spark of inspiration for Co-creation Hub’s collaboration with the Nigerian Institute of Medical Research (NIMR) on improving data integrity came from NimCure, a project conducted in 2018 with NIMR^3^ to develop a digital tool to support TB data management and promote adherence to TB therapy.

Research findings showed that individuals were providing inaccurate data on paper forms, leading to the inability to track patients who tested positive for TB. The most troubling example of the TB’s system’s inefficiency was when 2 patients who had both tested positive for TB could not be contacted with their test results due to inaccurate contact information. These 2 confirmed and contagious TB cases could not be reached either for treatment or for isolation due to poor data collection at the registration point.

This observation inspired the team to further explore the challenges with data collection and management process for TB patients. The team engaged with key stakeholders in the design process, which allowed the value generated to convince the stakeholders to rethink the way they collected data and how to improve that process.

To avoid similar data integrity issues, especially as new patients were recruited for the Nimcure medical trial, the design process was expanded to accommodate a reconstruction of the directly observed treatment center’s data collection system, thereby strengthening the integrity of data collected.

This system would later become the basis for first developing digital form at the COVID-19 test center to improve data collection and data integrity and later the outbreak management system to improve sample collection and management.

## OUR DESIGN APPROACH

### Discovery

Beginning in February 2020, during the discovery stage, a team from Co-creation Hub (CcHub) focused on observing and understanding the initial data collection problems at the drive-through center, as well as framing and reframing the problem given all the information gathered during this stage. Although the center was using an early format of a digital form, the team identified issues related to lack of prioritization, lack of accountability, a lack of direct consequence for inaccurate data, and misinterpretation of existing data. Additionally, because of a lack of quality control and limited workforces, the data integrity issues were amplified in dealing with the pandemic.

By starting with understanding what we did not know at the test center and what existing knowledge we had based on our experience with NimCure,^7^ we could reframe the problem around data integrity issues and ask whether these issues could be addressed by improving the initial digital form so that it enforced data entry compliance and quality control and providing a feedback mechanism so health care workers could understand the importance of quality data.

### Data Collection

In March and April 2020, our research and observations showed that the most prominent data integrity issues at the test center were accuracy, validity, and completeness (Table 1).^8^ These elements of data integrity became the primary indicators for measuring the success of the intervention. Interviews with NIMR personnel and review of the test center’s historic data helped to identify some specific instances of data integrity issues such as missing, incomplete, or inaccurate data. In some cases, inaccurate data, such as wrong addresses and phone numbers, were entered. In cases in which the addresses and phone numbers were correct, there were an overwhelming number of inconsistencies in the format in which they were stored.

**TABLE 1.** Co-Creation Hub Research and Observations Conducted at Nigerian Institute of Medical Research on Data Collection and Data Integrity

**Method**	**When**	**How**	**Outcome**
Guided tour and observation	February 2020 Before the first solution	The NIMR team led 2 members of the CcHUB team on a guided tour of the facility, and their entire data collection process was observed.	Identification of gaps and inconsistencies with the data collection and management process.
Secondary research	February 2020 At the inception of the new identified challenge (Improving the integrity of data collected)	The team explored recent publications, news articles, and case studies that focused on health care data management to identify some common challenges, approaches, and theories.	An understanding of the most important elements of data integrity in health care.
Observation	March 2020 During and after the secondary research	The team (CcHub and Lifebank) spent more time at the NIMR facility to explore their data collection and management process.	Identification of other inconsistencies with the data collection and management process.
Process mapping	April 2020 Before the drive-through COVID-19 test center	A co-creation session was held with the NIMR team to brainstorm on developing the process of operation for the drive-through COVID-19 test center that was to be based within the NIMR facility.	Process flow of the operations of the test center.
Co-creation sessions	April 2020 Before the drive-through COVID-19 test center	A series of subsequent co-creation sessions were held with members of both the NIMR and Lifebank administrative team. There were 5 virtual brainstorming sessions where participants co-created potential ideas for how to automate the process of the operation at the COVID-19 test center.	Up to 20 automation feature ideas that addressed data quality, crowd management, ease of communication, and many other administrative issues.
Survey and behavioral study	August-September 2020 After the launch of the COVID-19 test center and the digital system	The team conducted behavioral research in April to provide guidance for health administrators and policy makers on the factors that enhance the effective utilization of structures and platforms for service delivery. The study included 566 patients who had been invited for a COVID-19 test through the digital platform.	Unpublished data The findings also contributed to further development of the solution.

Abbreviations: CcHub, Co-creation Hub; COVID-19, coronavirus disease; NIMR, Nigerian Institute of Medical Research.

We conducted secondary research and a literature review to gather information on the characteristics of data integrity, how they apply to health care data, and the history of data integrity issues in health care. We aimed to identify whether these characteristics and issues applied to the test center’s data collection system and if measures were in place to strengthen data collection where it was weak (Table 2).

**TABLE 2.** Characteristics of Data Integrity Considered as They Relate to the NIMR COVID-19 Test Center

**Characteristics**	**Definition**
Completeness	Completeness refers to the availability of all relevant and required information. For example, a patient’s first and last name and address are required, but the middle initial and state or origin are optional. Data can be complete even if optional data are missing.
Accuracy	The degree to which data correctly describes the "real world" object or event being described. In the case of TB cases, the phone numbers provided did not reflect the patients’ phone numbers in the real world and as such were not accurate.
Timeliness	Timeliness considers how up-to-date the information is and how well it can be used for real-time reporting. The need to still transcribe data collected on paper forms at the point of registration creates a delay in real-time reporting.
Validity	Validity measures whether a value conforms to a preset standard, format, or syntax. Patient information like gender, weight, test results, etc. can easily be recorded with the wrong syntax. For example, recording weight in tons as opposed to pounds.
Uniqueness	Uniqueness is a measure of duplication of items in a data set. This measures how often 1 or more patients have had their data duplicated and listed as a separate entity.
Security	The protection of a patient’s data against unauthorized access or corruption is necessary to ensure data integrity. Specific patient data like test results and first line addresses are critical and need to be protected from authorized access.

### Design

In April 2020, we used the insights gathered from the discovery stage, found themes in the data, and used co-creation workshops together with stakeholders to design a solution that is relevant, applicable, and sustainable.

The Co-creation Hub’s NimCure experience encouraged the team to use the HCD approach to engage key stakeholders early in the co-creation process for an outbreak management system (Table 3).

**TABLE 3.** Relevant Stakeholders Engaged in the Human-Centered Design Approach of a Digital System in Nigeria

**Stakeholder**	
Patients	This included any suspected or high-risk persons who may be experiencing symptoms of the virus or may have been in contact with confirmed or probable cases and want to get tested or are required to get tested.
Health Facilities and Test Center Administrators	These were either specialized COVID-19 test centers or general health care facilities that have the capacity to run a significantly high number of tests daily to receive insights from other health care administrators that may benefit the solution focused on COVID-19. A key stakeholder in this category is NIMR, and in the case of this project, they provide all the necessary testing tools and infrastructure required to run the drive-through test center. NIMR personnel also served as the center administrators and lab managers. The role of the center administrators was to manage the drive-through system and oversee the collection of test samples. The lab managers were NIMR scientists responsible for running the tests, validating, and reporting results to the relevant authorities.
Lifebank	Health care technology and logistics company that is known for applying technology in solving health care problems. Lifebank took on the role of digital platform administrators and managed activities such as contacting patients, sending invites, while also supervising the use of the platform by other stakeholders (NIMR and patients) during the design process. As delivery partners for the test center, it was essential to engage NIMR and Lifebank teams in the design process, particularly in brainstorming sessions and product testing to encourage their buy-in and drive equity.
Government Institutions and Policy Makers	Timely and accurate data is critical in aiding government institutions better make strategic and timely decisions regarding the pandemic. The NCDC is the country’s public health institute responsible for the preparedness, detection, and response to infectious disease outbreaks and public health emergencies. As Nigeria’s foremost public health institute, data on all tests conducted daily were reported to NCDC representatives on the ground. Insights gathered from this data helped to coordinate national public health responses and policy making. The NCDC personnel were indirectly engaged through our partnership with NIMR to understand the most important data types and formats to them.
The Research Community and the General Public	The importance of accurate, complete, and timely data for research in public health cannot be underestimated as the insight gathered by the research community contributes to the body of knowledge available for the general public and policy makers. The system strengthens the integrity of data collected and analyzed by researchers which will, in turn, ensure accurate insights and better understanding and management of the pandemic.

Abbreviations: COVID-19, coronavirus disease; NCDC, Nigeria Center for Disease Control; NIMR, Nigerian Institute of Medical Research.

The engagement of the relevant stakeholders early in the design process contributed to increased equity, sustainability, and long-term impact by ensuring there is a strong buy-in and ownership from key stakeholders, especially the patients, public health facilities, and government institutions. An HCD approach puts the end users at the center of the process and solution. Engaging stakeholders allowed an easy entry point to apply the NimCure model to COVID-19 test center, which is usually a difficult step in the process. The design process was the spark that allowed the ecosystem players to use design and technology to find solutions during the COVID-19 pandemic.

The engagement of the relevant stakeholders early in the design process contributed to increased equity, sustainability, and long-term impact by ensuring there is a strong buy-in and ownership from key stakeholders.

The team at NIMR was engaged through a series of virtual co-creation sessions to brainstorm ideas on how to maximize the efficiency of the test center and ensure data integrity. Co-creation workshops also were held with a mix of the stakeholders to allow for diverse perspectives when deep diving into the problem that exists and potential solutions for it. In addition, each idea was run through storyboarding exercises that allowed for evaluation of the idea according to design principles, desirability, feasibility, and viability. These sessions allowed for various tweaks on the technology so that the different layers in the solution were deeply thought about, linked, and improved on. The entire journey from testing to reporting test results and allowing for tracing was thought about as an entire experience rather than parts in a silo.

Identifying the specific data integrity problems that existed at the test center sparked the design process to improve the digital form for registering patients before getting tested to ensure that only accurate, valid, and complete data were collected by including key features such as repeat or related-case flagging, phone number verification, and address validation. Additionally, the experience of the successful implementation of the digital form for NimCure allowed stakeholders to easily see and understand the need for data integrity and automated data collection.

The result of these design sessions inspired the idea for the NIMR staff to allow automated digital data collection and testing sample management at the drive-through center. This center became the first test center in Lagos state with automated data collection. The COVID-19 drive-through test center was unique from the NimCure TB directly observed treatment center because during the pandemic more patients took the initiative to get tested compared to the patients being referred to the test center by primary health care physicians. With COVID-19 being a highly contagious disease, there was an urgency in communicating the test results, and all test results had to be shared with the Nigerian Center for Disease Control daily.

## THE OUTBREAK MANAGEMENT SYSTEM

The design approach inspired the deployment of an automated data collection and management system based on the one used for NimCure for TB management. Through the engagement of all the relevant stakeholders during multiple rounds of iteration, this simple solution evolved into a practical digitized data management system that promotes data integrity and helps test centers more efficiently manage data.

Unique value proposition: By automating and streamlining data collection, the outbreak management system makes it easier and faster to manage data. The system retrieves information on demography, symptoms, pre-existing conditions, recent contact, and travel history to identify and triage high-risk cases that require testing. Some of the system’s key features are described here.

1. **Case reporting form:** People who think that they might have the virus and want to get tested can communicate their symptoms and request to be tested by completing an online digital form. Prospective patients submit the form to allow necessary information to be gathered faster and recorded more accurately.
2. **Triaging/eligibility indication:** The system automates the screening and triaging process based on the Nigerian Center for Disease Control case criteria to ensure that test centers’ capacity and resources are directed first toward cases with the highest risk level.
3. **Phone number verification:** This feature runs every phone number submitted on the platform through a nationwide database to confirm existence and validity.
4. **Address validation:** Using Google maps application programming interface, the system can validate every address provided to confirm that they indeed exist on the map.
5. **Access control:** The classification of users with specific privileges helps maintain data protection by ensuring data can only be accessed and modified by appropriate and qualified users.
6. **Privacy:** A unique identification code ensures the privacy of patients’ data.
7. **Contact tracing data**: The system collects a list of persons that each case may have been in recent contact with in the past to understand how the individual contracted the virus as well as who they may have been in contact with since the possible contracting point. These data are collected to support contact tracing for positive patients.
8. **Duplicate entry restriction:** Repeat or duplicate entries are detected and restricted based on name, phone number, email, and date of birth. This helps to ensure that all submissions are unique to a specific individual and that patients cannot make multiple submissions.

By automating and streamlining data collection, the outbreak management system makes it easier and faster to manage data.

Other features that were included during iteration were: test invitations, appointment scheduling, and result reporting.

**Figure:**
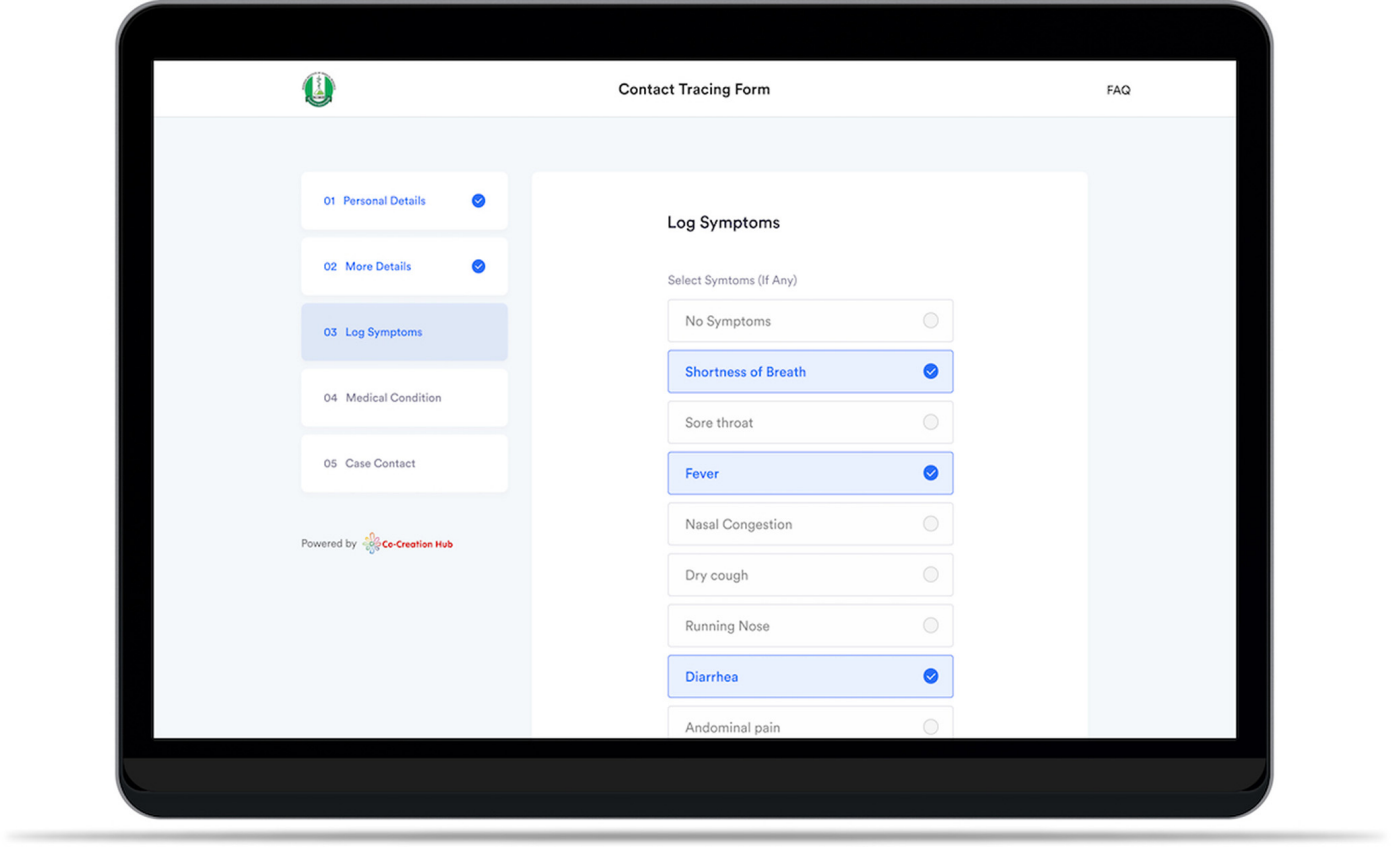
Screenshot of case reporting digital form co-created with Co-creation Hub and Lifebank in collaboration with the Nigerian Institute of Medical Research for the COVID-19 drive-through test center.

## RESULTS

In August 2020, the outbreak management system was tested with NIMR. By December 2020, the system had processed more than 30,000 reports with more than 75% of these reports considered eligible for testing based on their symptoms. With the use of this system, the test center has reported it is easier to collect and verify data, send appointment invitations to come to the center at a specific time, and report results, as well as faster to conduct center administrative processes.

According to NIMR, the system has significantly improved the quality of and use of the data collected through the test center, which is helping address the issue of data integrity. They have recorded improvements in confidentiality, completeness, accuracy, consistency, validity, and security of all data related to COVID-19 tests at the drive-through test center (Table 4).

**TABLE 4.** Early Results of Using the Digital System on Data Integrity in Lagos, Nigeria

**Characteristics**	**Feature**	**Result**
Completeness and consistency	Form validation	The platform has been able to achieve 100% completeness on all required fields.
Accuracy	Address validation	91% of all addresses (location) entered on the platform were validated. They all contain the state, local government area, and area details; and can be located on Google maps.
	Phone number validation and SMS communications	The platform was able to validate and verify 100% of all phone numbers.
Security and privacy	Strict access control	The platform has strict user access levels that allow us to ensure that only physicians and lab managers have access to test results.
	Data anonymization	To protect the patient's privacy, their data are anonymized for users who do not require them. For example, those who run tests and upload results do not have access to the patients’ personal information.
Timeliness	Real-time data collection and delivery	All data collected are made available on the dashboard in real-time with no delays.
Validity	Duplicate entries restriction	At the launch of the system, the platform received 75% unique submissions. However, after introducing the duplicate entry restriction feature in April 2020, this improved to 79% and by June 2020, we had up to 99% unique submissions.

The success of the system at the NIMR drive-through center has also inspired the automation of processes for other test centers around the country using HCD. The process was applied to engage stakeholders in other locations to deploy the solution for different test centers based on their mode of operation.

**Simple Testing with Lagos University Teaching Hospital:** The success of automating the drive-through test center inspired the automation of the Lagos University Teaching Hospital walk-in COVID-19 test center, which launched September 2020.

**Self-Testing with Lifebank:** The outbreak management system has also inspired the development of a self-testing kit. This version of the system enables the assignment of cases to select delivery persons who then deliver marked test kits to the patient's address. In addition, the features also enable the management of a chain of laboratories and test centers by assigning test samples to them, receiving results, and sending them to patients.

**Surveillance Visits with Ondo State COVID-19 Rapid Response Center:** Similar to the self-testing framework, new COVID-19 cases are assigned to surveillance experts who then visit the patients to confirm, record symptoms, and collect test samples. The system is currently being used to manage the center’s data collection, transfer, and other administrative processes from reporting via call centers to laboratory management.

## LESSONS LEARNED

### Engaging Stakeholders Facilitates Adoption and Diffusion of the System

The adoption of this system by the stakeholders has evolved from the early stage with the management of TB patients to COVID-19 testing. In many health facilities in Nigeria, paper records and forms are used to collect and store patient data, and the introduction of the digital form took a bit of adjustment. However, one strategic decision that was made to hasten the diffusion and adoption process was the intense engagement of stakeholders in the design process. As mentioned earlier, representatives of each stakeholder group were part of the co-creation sessions and were able to collectively design features that added more value for each group, thereby making the platform beneficial for them. Also, co-creating with the end users, the NIMR and Lifebank teams contributed to increasing equity and buy-in on the importance of proper usage of the platform. After deploying the solution, we conducted some training sessions to onboard the test center administrators and lab managers to the system and familiarize them with it.

### Lack of Research and Development Funding Can Hinder Innovation

A lack of ongoing research and development funds can make it quite difficult for organizations to expeditiously take on research and development projects that could lead to innovative products. The development of the outbreak management system was possible because of a pre-existing relationship between the research facility and CcHub that had been formed to build innovation through technology. This meant that both parties had to source the funds to support the project on their own.

### The Need for Scale

The goal of the outbreak management system in helping to maintain the integrity of the data collected at test centers is part of a bigger plan to consistently ensure that all distributed health care data points locally and regionally collect accurate, complete, and valid data. This gives rise to a need for scaling the product to as many facilities in as little time as possible.

## CONCLUSION

Ensuring integrity in health care data is imperative. The use of an outbreak management system to automate and streamline data collection and administrative processes in a COVID-19 test center in Nigeria proved to improve the integrity of data collected. Our experience shows that constant stakeholder engagement contributed to better adoption, a solution designed to ensure that data collected were complete and accurate, and inspiring the development of similar solutions.

## Notes

**Peer Reviewed**

## References

[1] Mate KS Bennett B Mphatswe W Barker P Rollins N. Challenges for routine health system data management in a large public programme to prevent mother-to-child HIV transmission in South Africa. PLoS One. 2009;4(5): e5483. 10.1371/journal.pone.0005483. 19434234 PMC2677154

[2] Anifalaje AA. Decentralisation and health systems performance in developing countries: impact of “decision space” on primary health care delivery in Nigeria. Int J Healthcare Delivery Reform Initiatives. 2009;1(1):25–48. 10.4018/JHDRI.2009010103

[3] Amoo OS Ohihoin AG Musa AZ. Implementation of a modified drive-through sampling strategy for SARS-CoV-2-the Nigerian experience. Pan Afr Med J. 2020;35(2):107. 10.11604/pamj.supp.2020.35.2.24319. 33282062 PMC7687503

[4] Salako AO Amoo OS Odubela OO. Prevalence and clinical characteristics of coronavirus disease 2019 seen at a testing centre in Lagos Nigeria. West Afr J Med. 2021;38(1):54–58. 33463708

[5] Bazzano AN Martin J Hicks E Faughnan M Murphy L. Human-centred design in global health: a scoping review of applications and contexts. PLoS One. 2017;12(11):e0186744. 10.1371/journal.pone.0186744. 29091935 PMC5665524

[6] Design for Health. Accessed October 4, 2021. www.designforhealth.org

[7] CcHub and NIMR launch NimCure—a digital tool to promote adherence to treatment by TB patients. Co-creation Hub website. October 9, 2018. Accessed September 27, 2021. https://cchubnigeria.com/cchub-and-nimr-launch-nimcure-a-digital-tool-to-promote-adherence-to-treatment-by-tb-patients/

[8] Tijani B Amoo OS Adaramewa T. Care seeking behavior of citizens during pandemics: a case study of COVID-19 in Nigeria. Acta Scientific Microbiology. 2020;3(9):137–152. Accessed September 27, 2021. https://actascientific.com/ASMI/pdf/ASMI-03-0689.pdf

